# Multimorbidity and the primary healthcare perspective

**DOI:** 10.15256/joc.2016.6.84

**Published:** 2016-04-21

**Authors:** Hilde D. Luijks, Antoine L.M. Lagro-Janssen, Chris van Weel

**Affiliations:** ^1^Department of Primary and Community Care, Radboud University Medical Center, Nijmegen, The Netherlands; ^2^Department of Health Services Research and Policy, Australian National University, Canberra, ACT, Australia

**Keywords:** multimorbidity, comorbidity, family medicine, primary healthcare, diabetes mellitus, family physician

The ageing population is marked by an increase in chronic health problems, raising concerns over the feasibility of healthcare systems and their financial capabilities [1,2]. A central point here is the growing rate of multimorbidity, i.e. the coexistence of multiple chronic conditions in a given individual [3].

The concept of multimorbidity conflicts with the ‘single-disease model’, around which healthcare, medicine and health research are traditionally organized. This model has dominated healthcare, research and education for so long that it is only recently that multimorbidity is being presented as a demographic feature.

Multimorbidity requires a paradigm shift away from this single-disease model of patient management; a shift that is now increasingly recognized and adopted, albeit at a slow pace. However, the reality in primary healthcare is already somewhat different. Primary healthcare, in its comprehensive approach to all health problems in all individuals at all disease stages and phases of life, has a long experience in dealing with individuals experiencing a range of health problems [4], including chronic health problems as reported in the literature [5–7]. These reports indicate that multimorbidity is substantial, with about a third of the (primary healthcare) population affected; this prevalence is in line with those reported in more recent studies from other countries [8–12].

Family physicians (FPs) can be regarded as experts in dealing with both comorbidity and multimorbidity in primary care; parallel to the expertise of geriatricians in secondary care. It is a logical assumption that empiricism and experience will yield original and innovative approaches to inform patient care. For this reason, we performed a series of studies to evaluate primary healthcare data and the experiences of FPs in managing comorbidity and multimorbidity [13–18]. The following is a summary of some of our findings and experiences where our use of the term comorbidity was aligned with the recent definition provided by Ramond-Roquin and Fortin: “comorbidity refers to an additional condition(s) in an individual who has a given index disease…” [3].

## Patterns of comorbidity: a case study of diabetes mellitus

The high prevalence of multimorbidity in the population can often mean that patient-group- or disease-specific patterns are concealed. We analysed the prevalence and incidence density of chronic comorbid diseases in a representative primary healthcare cohort of patients with recently diagnosed type 2 diabetes mellitus [13].

As expected, a high proportion of this cohort had chronic health problems other than diabetes: hypertension (38%) and chronic venous insufficiency (21%) were highly prevalent, as were chronic functional somatic symptoms (19%), hearing loss (14%), urinary incontinence (13%), angina pectoris (12%), osteoarthritis of the knee (12%), chronic obstructive pulmonary disease (11%), prostatic hyperplasia (10%), and atrial fibrillation/flutter (9%). Cardiovascular diseases (CVD) accounted for a substantial proportion of the comorbidity, which is to be expected given that diabetes is a risk factor for ischaemic CVD. In this respect, our study confirmed the risk relationship between diabetes and ischaemic CVD. However, we found that a large proportion of the comorbidity was pathophysiology unrelated to the development of diabetes and outnumbered cardiovascular comorbid conditions; a finding that has also been reported elsewhere [19–22]. These findings highlight that the diabetes population is heterogeneous in terms of comorbidity, and only a few patients had no comorbidity at all. This makes it almost impossible to characterize ‘the typical diabetes patient’.

## Types of comorbidity: concordant and discordant

From the findings in the diabetes cohort described above, we identified two types of comorbidity, concordant and discordant, in line with the definition proposed by Piette and Kerr [23]. Concordant comorbidity is related to the pathophysiology of the index disease and shares a common treatment approach, thereby consolidating patient care. In contrast, discordant comorbidity is unrelated to the pathophysiology of the index disease and does not share the same treatment approach. Instead, it requires separate management of the distinct diseases; an approach that increases the risk of conflicting strategies, polypharmacy, interactions, and side effects. Discordant comorbidity is probably better characterized as multimorbidity [3].

## Impact of comorbidity

Comorbidity may have an important impact on long-term prognosis and outcome of care. We explored this in the cohort of patients with diabetes, using intermediate markers of outcome of care: systolic blood pressure (SBP) and glycaemic control (HbA1c), as defined in the Dutch College of General Practitioners practice guidelines on type 2 diabetes [24]. Surprisingly, it was not the number of comorbid diseases that had a negative influence on these parameters of long-term diabetes control; instead, it was the specific comorbidity. For example, patients with comorbid musculoskeletal disease had higher HbA1c values 5 years after the diagnosis of diabetes, whereas patients with comorbid CVD had sustained elevated levels of SBP.

Elevated SBP was also often seen in patients with diabetes and comorbid chronic obstructive pulmonary disease (COPD) [15], a disease common in the (elderly) population [25] and particularly prevalent (11%) in our cohort of patients with diabetes [13]. In this group of patients with diabetes and comorbid COPD, we analysed the effects of socioeconomic status and body mass index and concluded that socioeconomic status was a strong determinant of an unfavourable outcome in SBP [15].

## Patient care: challenges and empirical solutions

A series of focus groups were held with FPs to explore their experiences, approaches and strategies in their care of patients with multiple chronic health problems [16,17].

FPs experienced comorbidity as a challenge to providing optimal patient care. In particular, the combined presence of somatic and mental health conditions was perceived as a difficult combination, increasing the difficulty of diagnosis and treatment of both somatic and mental health conditions as symptom presentation and treatment adherence may be altered. One condition may be experienced as ‘overshadowing’ another. In general, comorbidity did bring with it the risk of fragmentation of care through separate and uncoordinated disease-directed interventions [16].

The Dutch College of General Practitioners has guidelines for a substantial number of chronic conditions [26], and the application of these guidelines in the care of patients with comorbidity was an issue for FPs. Although FPs were positive about the professional guidance the guidelines offered, their general opinion was that guidelines alone were insufficient to address the needs of patients with multimorbidity and the associated complexity [17]. In particular, preventative interventions were often seen as an inappropriate burden to patient care. There were more general concerns about the role of guidelines in prescribing the content of care rather than in providing advice on possible evidence-based directions. FPs stressed the importance of an approach that combined the best available evidence with their clinical experience and knowledge of the person with the disease to optimize the management of multimorbidity.

In the focus group discussions, a clear strategy emerged of how FPs met the challenges of patients with multimorbidity [16,17]. Their personal relationship of trust built over time with the patient and family was seen as their operational clinical basis. First and foremost, they invested in this relationship and made sure that it was preserved, particularly when there was uncertainty or differences in opinion on the best treatment options. A person-centred approach with shared decision making was seen as the best way to handle the pitfalls of managing multimorbidity. This person-centredness made it possible to place intrinsic medical considerations – including guideline recommendations – in a broader individual perspective for decision making.

## Conclusions and recommendations

Our studies confirm the high prevalence of multimorbidity in the primary healthcare setting and the variation in comorbid conditions between patients with the same index disease. For the most part, comorbid conditions were discordant, showing comorbidity to be a personal rather than a disease-related characteristic. Our findings were based on type 2 diabetes mellitus as the index condition, and exploring the variation in comorbidities with other common chronic conditions is warranted.

Our findings highlight the importance of a person-centred approach by FPs. This may be due to the fact that in coping with the clinical challenges of caring for patients with multimorbidity, practice is ahead of science. Future research should build on the empiricism that practice has built over the years: to support the development and maintenance of trusting relationships over time and decision making that shares the wisdom of the patient and FP professional.

This also has implications for the structure of healthcare: enabling personal relationships with patients and awarding working over time in response to individual needs. This is particularly relevant for primary healthcare as, in the community setting, health and well being are linked. Our data reveal that most individuals experience multiple chronic diseases. Add to that the social and economic problems people are facing and it is clear that hardly anyone can be characterized by a single (health) problem.
